# PIK3CA-activating mutations and chemotherapy sensitivity in stage II–III breast cancer

**DOI:** 10.1186/bcr1984

**Published:** 2008-03-27

**Authors:** Cornelia Liedtke, Luca Cardone, Attila Tordai, Kai Yan, Henry L Gomez, Luis J Barajas Figureoa, Rebekah E Hubbard, Vicente Valero, Eduardo A Souchon, W Fraser Symmans, Gabriel N Hortobagyi, Alberto Bardelli, Lajos Pusztai

**Affiliations:** 1 Department of Breast Medical Oncology, University of Texas M. D. Anderson Cancer Center, Houston, TX, USA; 2 Department of Gynecology and Obstetrics, University Hospital Münster, Münster, Germany; 3 Laboratory of Molecular Genetics, The Oncogenomics Center, Institute for Cancer Research and Treatment (IRCC), University of Torino Medical School, Candiolo, Italy, FIRC Institute of Molecular Oncology (IFOM), Milan, Italy; 4 Laboratory of Molecular Genetics, Institute of Hematology and Immunology, National Medical Center, Budapest, Hungary; 5 Department of Biostatistics, University of Texas M. D. Anderson Cancer Center, Houston, TX, USA; 6 Instituto Nacional de Enfermedades Neoplasicas, Lima, Peru; 7 Departamento de Ginecología Oncológica, Gineco-Obstetricia, Instituto Mexicano del Seguro Social, Guadalajara, Jalisco, Mexico; 8 Department of General Surgery, Lyndon B. Johnson Hospital, Houston, TX, USA; 9 Department of Pathology, University of Texas M. D. Anderson Cancer Center, Houston, TX, USA

## Abstract

**Introduction:**

*In vitro *evidence suggests that PIK3CA (phosphatidylinositol 3-kinase, catalytic, alpha polypeptide) activation may be associated with altered chemotherapy sensitivity in cancer.

**Methods:**

Tumor DNA from 140 patients with stage II–III breast cancer undergoing neoadjuvant chemotherapy was sequenced for PIK3CA mutations on exons 1, 9, and 20. Mutation status was correlated with clinical/pathological parameters and chemotherapy response as (a) pathological complete response (pCR) versus residual cancer or (b) quantitative residual cancer burden (RCB) scores, including stratification for estrogen receptor (ER) expression status, type of chemotherapy, and by exons.

**Results:**

Twenty-three patients (16.4%) harbored a PIK3CA mutation, with 12, 11, and 0 mutations located in exons 9, 20, and 1, respectively. PIK3CA exon 9 mutations were more frequent among node-negative (52% versus 25%; *P *= 0.012) than node-positive tumors, particularly among ER-positive tumors. pCR rates and RCB scores were similar among patients with the wild-type and mutant PIK3CA genes, even after stratification by ER status, chemotherapy regimen (anthracycline versus anthracycline plus paclitaxel), or exon.

**Conclusion:**

PIK3CA mutations are not associated with altered sensitivity to preoperative anthracycline-based or taxane-based chemotherapies in ER-positive and ER-negative breast tumors. In this study, PIK3CA mutation was associated with a decreased rate of node-positive disease, particularly among ER-positive tumors.

## Introduction

Phosphoinositol 3-kinase (PI3K) is a heterodimer that is composed of a p85 regulatory and a p110 catalytic subunit (coded for by the PIK3CA [PI3K, catalytic, alpha polypeptide] gene) [1,2]. PI3K activity controls multiple cellular functions through its second messenger, 3,4,5'-phosphatidylinositol trisphosphate, and its downstream targets, including the serine/threonine protein kinases Akt and mammalian target of rapamycin (mTOR) [3]. Activation of the PI3K/Akt pathway is involved in the regulation of cell proliferation and suppression of apoptosis [4]. Activating mutations in the catalytic subunit are oncogenic *in vivo *[5]. Almost all activating mutations (>90%) in human tumors occur in exons 9 (helical domain E542K and E545K) and 20 (kinase domain H1047R); the remainder seem to be distributed evenly over the entire PIK3CA coding sequence. Activating mutations induce a gain of function that results in constitutive signaling through the PI3K/Akt and mTOR pathways [6]. PIK3CA is frequently mutated in different human tumors, including head and neck, cervical, gastric, lung, and breast tumors [7]. In breast cancer, PIK3CA mutations occur in approximately 18% to 40% of human cases and are also observed in up to 50% of breast cancer cell lines [8-14].

*In vitro *evidence suggests that PIK3CA activation is associated with decreased sensitivity to several different chemotherapeutic agents, including paclitaxel, doxorubicin, or 5-fluorouracil [15,16]. The goal of this study was to examine whether there is a correlation between activating mutations in the catalytic subunit of PI3K and response to therapy in stage II–III human breast cancer treated with preoperative chemotherapy. We hypothesized that activation of this pathway through somatic mutations may be associated with decreased response to cytotoxic treatment and increased residual cancer volume after chemotherapy. We examined this potential effect separately for estrogen receptor (ER)-positive and for ER-negative breast tumors and also for anthracycline-based and anthracycline/paclitaxel-based chemotherapies. To our knowledge, this is the first breast cancer study to directly examine the association between PIK3CA mutation status and response to chemotherapy in breast cancer.

## Materials and methods

### Patient characteristics

The study population consisted of 140 patients who participated in a pharmacogenomic predictive marker discovery study at the University of Texas M. D. Anderson Cancer Center (MDACC) [17]. During this research, patients were asked to undergo pretreatment fine needle aspiration (performed with a 23- or 25-gauge needle) of the primary breast tumor. Cells from two or three passes were collected into vials containing 1 mL of RNAlater™ solution (Ambion, Inc., Austin, TX, USA) and stored at -80°C. All patients subsequently received 6 months of preoperative chemotherapy: 63 patients (45%) received six courses of 5-fluoruracil, doxorubicin (or epirubicin), and cyclophosphamide (FAC or FEC, respectively) chemotherapy, and 77 patients (55%) received 12 weekly courses of paclitaxel followed by four courses of 5-fluoruracil, doxorubicin (or epirubicin), and cyclophosphamide (TFAC or TFEC, respectively). None of these patients received preoperative treatment with trastuzumab, lapatinib, or endocrine therapy. All patients underwent modified radical mastectomy or lumpectomy and sentinel node dissection after completion of chemotherapy. All patients with ER-positive tumors subsequently received adjuvant endocrine therapy. Each patient gave informed consent to allow molecular analysis of her tumor, and this study was approved by the institutional review board of the MDACC. Patient characteristics are summarized in Table 1.

**Table 1:** Patient characteristics

		Number	Percentage
Pathological complete response (pCR) versus residual disease (RD)	RD	113	80.7
	pCR	24	17.1
	Unknown	3	-
Residual cancer burden	0	24	22.6
	I	7	6.6
	II	47	44.3
	III	28	26.4
	Unknown	34	-
Estrogen receptor (ER) status	ER^-^	62	44.3
	ER^+^	78	55.7
Progesterone receptor (PR) status	PR^-^	82	58.6
	PR^+^	58	41.4
HER2 status	HER2^-^	125	89.3
	HER2^+^	15	10.7
Grade	Grade 1–2	56	48.7
	Grade 3	59	51.3
	Unknown	25	-
Nodal status and T stage	N0	41	29.3
	N1	62	44.3
	N2	30	21.4
	N3	7	5.0
	T1	9	6.4
	T2	71	50.7
	T3	21	15.0
	T4	39	27.9
Ethnicity	Asian	3	2.1%
	Black	13	9.3%
	Hispanic	50	35.7%
	Caucasian	74	52.9%
Systemic therapy	FAC/FEC	63	45.0%
	TFAC/TFEC	77	55.0%
Median age (minimum-maximum), years		51 (28–73)	

FAC, 5-fluoruracil, doxorubicin, and cyclophosphamide; FEC, 5-fluoruracil, epirubicin, and cyclophosphamide; TFAC, paclitaxel followed by 5-fluoruracil, doxorubicin, and cyclophosphamide; TFEC, paclitaxel followed by 5-fluoruracil, epirubicin, and cyclophosphamide.

### Pathology assessment

ER expression status and progesterone receptor (PR) expression status were assessed by immunohistochemistry (IHC) (6F11; Novocastra Laboratories Ltd., Newcastle, UK) and human epidermal growth receptor 2 (HER2) status was assessed by either fluorescence *in situ *hybridization (FISH) or IHC as part of routine clinical care. ER positivity and PR positivity were defined as greater than 10% positive tumor cells with nuclear staining. HER2 positivity was defined as either HER2 gene amplification on FISH analysis (>2.0 CYP16/HER2 gene copy number ratio) or 3+ signal on IHC evaluation. Nuclear grade was assessed using modified Black's nuclear grading system. Pathological response was determined at the time of surgery by microscopic examination of the excised tumor and lymph nodes. Pathological complete response (pCR) was defined as no residual invasive cancer in either tumor or lymph nodes as opposed to residual disease (RD). Cases with *in situ *carcinoma in the absence of an invasive component were also included among the cases with pCR [18]. Cases with residual cancer (RD) represent a continuum of responses and it has long been recognized that the larger the residual cancer after preoperative chemotherapy, the worse the prognosis. We recently developed a method to quantify residual invasive cancer after preoperative chemotherapy on a continuous scale. This method combines the largest diameter of the invasive tumor, the percentage cellularity of the tumor, the number of lymph nodes involved, and the largest diameter of the nodal involvement into a residual cancer burden (RCB) score [19,20]. The RCB score correlates with survival and also can be used to define four distinct pathological response categories: RCB-0 (same as pCR), RCB-I (near pCR), RCB-II (moderate residual cancer), and RCB-III (extensive residual cancer). These RCB categories are predictive of long-term survival; patients who achieve RCB-I pathological response have overall and disease-free survival rates similar to those of patients achieving pCR (that is, RCB-0) whereas patients with RCB-III have a very poor prognosis, particularly if they have ER-negative disease [20].

### DNA isolation and mutation analysis

DNA was extracted from the flow-through of the RNA extraction step performed with a Qiagen RNEasy Mini Kit (#74104; Qiagen Inc., Valencia, CA, USA) using a Qiagen DNA extraction kit (#69504; Qiagen Inc.) according to the manufacturer's instructions. DNA concentration and purity were determined using a NanoDrop ND-1000 Spectrometer (NanoDrop Technologies, Wilmington, DE, USA).

Sequences for all annotated exons and adjacent intronic sequences containing the kinase domain of the PIK3CA gene were extracted from the Celera (Rockville, MD, USA) [21] or public [22] draft human genome sequences. Primers for polymerase chain reaction (PCR) amplification and sequencing were designed using the Primer3 program [23] and were synthesized by MWG (High Point, NC, USA) or Integrated DNA Technologies, Inc. (Coralville, IA, USA). PCR amplification and PIK3CA sequencing were performed using a 384-capillary automated sequencing apparatus (Spectrumedix, State College, PA, USA). Sequence traces were assembled and analyzed to identify potential genomic alterations using the Mutation Surveyor software package (SoftGenetics, LLC, State College, PA, USA). Primer sequences and conditions for PCR amplification and sequencing have been reported previously [7,24]. Exon-specific and sequencing primers were synthesized by Invitrogen Corporation (Carlsbad, CA, USA). Purified PCR products were sequenced using a BigDye® Terminator version 3.1 Cycle Sequencing Kit (Applied Biosystems, Foster City, CA, USA) and analyzed with a 3730 ABI capillary electrophoresis system. Mutational analysis was carried out in the laboratory of author AB at the University of Torino [24].

### Statistical analysis

The correlation between PIK3CA mutation status and dichotomous clinical/pathological parameters was examined by means of the chi-square test. ER, PR, and HER2 receptor expression status (positive versus negative), nuclear grade (1/2 versus 3), and lymph node status (negative versus positive) were considered as dichotomous variables. Tumor size (T0–T4) and patient ethnicity (Asian, Black, Hispanic, and Caucasian) were treated as categorical variables, and patient age was treated as a continuous variable. Pathological response was examined as both a dichotomous variable comparing pCR versus all RD and as an ordinal categorical variable (RCB-0, -I, -II, and -III). The associations between continuous variables and PIK3CA mutation status were determined using the unequal variance *t *test. A *P *value of less than 0.05 was considered significant.

## Results

### PIK3CA mutation status of study cohort

The mutational status of the PIK3CA gene was assessed in all 140 tumors by direct sequencing of the gene regions encoding the helical domain (exon 9) and the catalytic domain (exon 20) of the PIK3CA gene. Tumor DNA was used from needle aspiration biopsy material that contains 75% to 90% cancer cells. One hundred seventeen tumors (83.6%) had the wild-type PIK3CA gene and 23 patients had an activating mutation in the PIK3CA gene (16.4%). Among the cases with a PIK3CA mutation, 12 had a missense mutation in exon 9 (8 E545K type, 3 E542K type and 1 Q546R) and 11 cases had a mutation in exon 20 (all but 2 were H1047R). Table 2 lists all of the detected mutations. We also examined mutations in exon 1 but no mutation was found in any of the cases.

**Table 2:** Types of PIK3CA mutations that were detected

Patient	Treatment category	Exon 9	Exon 20
1	FAC/FEC	E545K	
2	FAC/FEC		H1047R
3	FAC/FEC		H1047R
4	FAC/FEC	E542K	
5	FAC/FEC	E545K	
6	FAC/FEC		H1047R
7	FAC/FEC	E545K	
8	FAC/FEC	E545K	
9	FAC/FEC		H1047R
10	FAC/FEC	E545K	
11	FAC/FEC	E545K	
12	FAC/FEC	E545K	
13	TFAC/TFEC	Q546R	
14	TFAC/TFEC		H1047T
15	TFAC/TFEC		H1047R
16	TFAC/TFEC		H1047R
17	TFAC/TFEC		H1047R
18	TFAC/TFEC	E545K	
19	TFAC/TFEC	E542K	
20	TFAC/TFEC	E542V	
21	TFAC/TFEC		G1049R
22	TFAC/TFEC		H1047R
23	TFAC/TFEC		H1047R

Exon 1 mutations were also examined but no mutations were found. FAC, 5-fluoruracil, doxorubicin, and cyclophosphamide; FEC, 5-fluoruracil, epirubicin, and cyclophosphamide; PIK3CA, phosphatidylinositol 3-kinase, catalytic, alpha polypeptide; TFAC, paclitaxel followed by 5-fluoruracil, doxorubicin, and cyclophosphamide; TFEC, paclitaxel followed by 5-fluoruracil, epirubicin, and cyclophosphamide.

### Correlation between PIK3CA mutation status and clinical/pathological variables

When all of the cases were considered together, PIK3CA mutation was significantly associated with lymph node-negative status; 52% of mutant cases were node-negative compared with 25% among the wild-type cases (*P *= 0.012). There was also a trend for increased frequency of PIK3CA mutations in older women. The median age of patients with a PIK3CA mutation was 56 years compared with 51 years for the wild-type cases (*P *= 0.0535). No other clinical or pathological factor was significantly associated with PIK3CA mutation status (Table 3). In a multivariate model that included patient ethnicity, tumor grade (1/2 versus 3), tumor size, nodal stage, ER, PR, and HER2 status, patient age as well as response to chemotherapy, nodal status remained independently associated with PIK3CA mutation (*P *= 0.029).

**Table 3:** Correlation between PIK3CA mutation status and clinical variables

		PIK3CA wild-type	PIK3CA mutated	*P *value^a^
Pathological complete response (pCR) versus residual disease (RD)	RD	95 (83%)	18 (82%)	1.000
	pCR	20 (17%)	4 (18%)	
	Unknown	2	1	-
Residual cancer burden	0	20 (22.0%)	4 (26.7%)	0.121 (0.166^b^)
	I	7 (7.7%)	0 (0%)	
	II	37 (40.7%)	10 (66.7%)	
	III	27 (29.7%)	1 (6.7%)	
	Unknown	26	8	-
Estrogen receptor (ER) status	ER^-^	54 (46%)	8 (35%)	0.365
	ER^+^	63 (54%)	15 (65%)	
Progesterone receptor (PR) status	PR^-^	71 (60.7%)	11 (47,8%)	0.259
	PR^+^	46 (39.3)	12 (52,2%)	
HER2 status	HER2^-^	104 (89%)	21 (91%)	1.000
	HER2^+^	13 (11%)	2 (9%)	
Grade	Grade 1–2	46 (47%)	10 (56%)	0.612
	Grade 3	51 (53%)	8 (44%)	
	Unknown	20	5	-
Nodal status	Negative	29 (25%)	12 (52%)	0.012
	Positive	88 (75%)	11 (48%)	
Tumor size	T0	1 (1%)	1 (4%)	0.535
	T1	7 (6%)	0 (0%)	
	T2	59 (50%)	12 (52%)	
	T3	18 (15%)	3 (13%)	
	T4	32 (27%)	7 (30%)	
Ethnicity	Asian	2 (2%)	1 (4%)	0.505
	Black	11 (9%)	2 (9%)	
	Hispanic	40 (34%)	10 (43%)	
	Caucasian	64 (55%)	10 (43%)	
Median age (minimum-maximum), years		50 (28–73)	52 (42–73)	-

^a^Chi-square test. ^b^*P *value for comparison of residual cancer burden (RCB)-0 and RCB-I versus RCB-III. PIK3CA, phosphatidylinositol 3-kinase, catalytic, alpha polypeptide.

### Correlation between PIK3CA mutation status and clinical/pathological variables in ER-positive and ER-negative subgroups

We also examined the association between clinical and pathological parameters and PIK3CA mutation status in ER-negative (n = 62) and ER-positive (n = 78) tumors separately. No significant correlation was found between any clinical variable and PIK3CA mutation status among the ER-negative tumors. In contrast, among the ER-positive tumors, PIK3CA mutation status was significantly and inversely associated with nodal status. Patients with ER-positive tumors who were also positive for PIK3CA mutation had a higher incidence of node-negative disease (53% versus 22%; *P *= 0.025). No other clinical/pathological factor was associated with PIK3CA status in patients with ER-positive tumors (Table 4).

**Table 4:** Correlation between PIK3CA mutation and clinical variables in estrogen receptor (ER)-positive and ER-negative tumors

		Patients with ER-negative breast cancer			Patients with ER-positive breast cancer		
		
		PIK3CA wild-type (n = 54)	PIK3CA mutation (n = 8)	*P *value^a^	PIK3CA wild-type (n = 63)	PIK3CA mutation (n = 15)	*P *value^a^
Pathological complete response (pCR) versus residual disease (RD)	RD	38 (72%)	5 (71%)	1.000	57 (92%)	13 (87%)	0.617
	pCR	15 (28%)	2 (29%)		5 (8%)	2 (13%)	
	Unknown	1	1	-	1	-	-
Residual cancer burden	0	15 (34.1%)	2 (50.0%)	0.616 (0.527^b^)	5 (10.6%)	2 (18.2%)	0.221 (0.543^b^)
	I	3 (6.8%)	0 (0%)		4 (8.5%)	0 (0%)	
	II	15 (34.1%)	2 (50.0%)		22 (46.8%)	8 (72.7%)	
	III	11 (25.0%)	0 (0%)		16 (34.0%)	1 (9.1%)	
	Unknown	10	4	-	16	4	-
HER2 status	HER2^-^	47 (87%)	7 (88%)	1.000	57 (90%)	14 (93%)	0.617
	HER2^+^	7 (13%)	1 (12%)		6 (10%)	1 (7%)	
Grade	Grade 1–2	9 (20%)	2 (33%)	0.598	37 (71%)	8 (67%)	0.739
	Grade 3	36 (80%)	4 (67%)		15 (29%)	4 (33%)	
	Unknown	9	2	-	11	3	-
Nodal status	Negative	15 (28%)	4 (50%)	0.235	14 (22%)	8 (53%)	0.025
	Positive	39 (72%)	4 (50%)		49 (78%)	7 (47%)	
Tumor size	T0	0 (0%)	0 (0%)	0.937	1 (2%)	1 (7%)	0.715
	T1	4 (7%)	0 (0%)		3 (5%)	0 (0%)	
	T2	26 (48%)	4 (50%)		33 (52%)	8 (53%)	
	T3	10 (18%)	1 (12%)		8 (13%)	2 (13%)	
	T4	14 (26%)	3 (38%)		18 (28%)	4 (27%)	
Ethnicity	Asian	1 (2%)	0 (0%)	0.326	1 (2%)	1 (7%)	0.478
	Black	6 (11%)	2 (25%)		5 (8%)	0 (0%)	
	Hispanic	16 (30%)	4 (50%)		24 (38%)	6 (40%)	
	Caucasian	31 (57%)	2 (25%)		33 (52%)	8 (53%)	
Median age (minimum-maximum), years		51 (28–73)	56.5 (42–73)	-	50 (28–73)	52 (43–73)	

^a^Chi-square test. ^b^*P *value for comparison of residual cancer burden (RCB)-0 and RCB-I versus RCB-III. PIK3CA, phosphatidylinositol 3-kinase, catalytic, alpha polypeptide.

### Association between PIK3CA mutation status and pathological response to chemotherapy

We examined the correlation between PIK3CA mutation status and response to chemotherapy in all cases and after stratification by ER status. When all of the cases were considered together, there was no difference in pCR rate (pCR = extreme chemotherapy sensitivity) among the PIK3CA mutant (pCR = 18%) and wild-type (pCR = 17%) cases (Table 3). In ER-positive tumors, the pCR rates were 8% and 13% (*P *= 0.62) in tumors with wild-type and mutant PIK3CA, respectively. In ER-negative tumors, the pCR rates were 28% and 29% (*P *= 1.0) for the wild-type and mutant cases, respectively (Table 4). Next, we examined pCR rates by type of chemotherapy and PIK3CA mutation status. Sixty-three patients received neoadjuvant FAC/FEC chemotherapy and the pCR rates were 6% and 8% for the wild-type and mutant cases, respectively (*P *= 1.0). Seventy-seven patients received neoadjuvant TFAC/TFEC chemotherapy and the pCR rates were 27% and 30% for the wild-type and mutant cases, respectively (*P *= 1.0) (Table 5).

**Table 5:** Correlation between PIK3CA mutation status and response to neoadjuvant FAC/FEC or TFAC/TFEC chemotherapies

		FAC/FEC^a ^chemotherapy			TFAC/TFEC^a ^chemotherapy		
		
		PIK3CA wild-type (n = 51)	PIK3CA mutation (n = 12)	*P *value^b^	PIK3CA wild-type (n = 66)	PIK3CA mutation (n = 11)	*P *value^b^
Pathological complete response (pCR) versus residual disease (RD)	RD	48 (94%)	11 (92%)	1.000	47 (73%)	7 (70%)	1.000
	pCR	3 (6%)	1 (8%)		17 (27%)	3 (30%)	
	Unknown	-	-	-	2	1	-
Residual cancer burden	0	6 (17.6%)	1 (16.7%)	0.334 (0.474^c^)	14 (24.6%)	3 (33.3%)	0.438 (0.613^c^)
	I	2 (5.9%)	0 (0%)		5 (8.8%)	0 (0%)	
	II	16 (47.1%)	5 (83.3%)		21 (36.8%)	5 (55.6%)	
	III	10 (29.4%)	0 (0%)		17 (29.8%)	1 (11.1%)	
	Unknown	17	6	-	9	2	-

^a^For description, please refer to text. ^b^Chi-square test. ^c^*P *value for comparison of residual cancer burden (RCB)-0 and RCB-I versus RCB-III. FAC, 5-fluoruracil, doxorubicin, and cyclophosphamide; FEC, 5-fluoruracil, epirubicin, and cyclophosphamide; PIK3CA, phosphatidylinositol 3-kinase, catalytic, alpha polypeptide; TFAC, paclitaxel followed by 5-fluoruracil, doxorubicin, and cyclophosphamide; TFEC, paclitaxel followed by 5-fluoruracil, epirubicin, and cyclophosphamide.

It has been suggested that mutations in exon 9 may have different functional consequences than mutations in exon 20; therefore, we also tested the association between mutation type and response to chemotherapy [25]. There was no difference in pCR rates associated with mutation in either exon individually. However, in correlation analysis, nodal stage was associated with PIK3CA mutation status only for those in exon 9, with patients harboring an exon 9 mutation having an increased incidence of node-negative disease (66.7%) compared with patients with wild-type or other mutation types (24.8%; *P *= 0.023). Mutations in exon 20 were not significantly associated with any clinical or pathological parameter (Table 6).

**Table 6:** Correlation between PIK3CA mutation type and clinical variables, including pathological response to chemotherapy

		Exon 9 mutation status			Exon 20 mutation status		
		
		Wild-type (n = 117)	Mutation (n = 12)	*P *value^a^	Wild-type (n = 117)	Mutation (n = 11)	*P *value^a^
Pathological complete response (pCR) versus residual disease (RD)^b^	RD	95 (82.6%)	10 (83.3%)	0.656	95 (82.6%)	8 (80.0%)	0.689
	pCR	20 (17.4%)	2 (16.7%)		20 (17.4%)	2 (20.0%)	
	Unknown	2	-	-	2	1	-
Residual cancer burden	0	20 (22.0%)	2 (25.0%)	0.524 (0.513^c^)	20 (22.0%)	2 (28.6%)	0.243 (0.492^c^)
	I	7 (7.7%)	0 (0%)		7 (7.7%)	0 (0%)	
	II	37 (40.7%)	5 (62.5%)		37 (40.7%)	5 (71.4%)	
	III	27 (29.7%)	1 (12.5%)		27 (29.7%)	0 (0%)	
	Unknown	26	4		26	5	
HER2 status	HER2^-^	104 (88.9%)	11 (91.7%)	0.617	104 (88.9%)	10 (90.9%)	0.659
	HER2^+^	13 (11.1%)	1 (8.3%)		13 (11.1%)	1 (9.1%)	
Grade	Grade 1–2	46 (47.4%)	4 (50.0%)	0.561	46 (47.4%)	6 (60.0%)	0.112
	Grade 3	51 (52.6%)	4 (50.0%)		51 (52.5%)	4 (40.0%)	
	Unknown	20	4	-	20	1	-
Nodal status	Negative	29 (24.8%)	8 (66.7%)	0.023	29 (24.8%)	4 (36.4%)	0.761
	Positive	88 (75.2%)	4 (33.3%)		88 (75.2%)	7 (64.6%)	
Tumor size	T0	1 (0.9%)	1 (8.3%)	0.322	1 (0.9%)	0 (0%)	0.854
	T1	7 (6.0%)	0 (0%)		7 (6.0%)	0 (0%)	
	T2	50 (50.4%)	6 (50.0%)		59 (50.4%)	6 (54.5%)	
	T3	18 (15.4%)	2 (16.7%)		18 (15.4%)	1 (9.1%)	
	T4	32 (27.4%)	3 (25.0%)		32 (27.4%)	4 (36.4%)	
Ethnicity	Asian	2 (1.7%)	0 (0%)	0.544	2 (1.7%)	1 (9.1%)	0.290
	Black	11 (9.4%)	0 (0%)		11 (9.4%)	2 (18.2%)	
	Hispanic	40 (34.2%)	6 (50.0%)		40 (34.2%)	4 (36.4%)	
	Caucasian	64 (54.7%)	6 (50.0%)		64 (54.7%)	4 (36.4%)	
Median age (minimum-maximum), years	50 (28–73)	53 (42–72)	0.313	50 (28–73)	50 (28–73)	0.084	

^a^Chi-square test. ^b^TFAC (paclitaxel followed by 5-fluoruracil, doxorubicin, and cyclophosphamide) or FAC (5-fluoruracil, doxorubicin, and cyclophosphamide) chemotherapies combined. ^c^*P *value for comparison of residual cancer burden (RCB)-0 and RCB-I versus RCB-III. PIK3CA, phosphatidylinositol 3-kinase, catalytic, alpha polypeptide.

RCB response category was available for 106 patients and this provided an opportunity to correlate PIK3CA mutation with graded pathological response. We compared PIK3CA mutation frequency in all four RCB categories and also in the two extreme response groups: RCB-0/I (highly chemotherapy-sensitive cases) versus RCB-III (highly chemotherapy-resistant tumors). No significant association was found between RCB response categories and PIK3CA mutation status in either analysis (*P *= 0.121 and 0.166, respectively) (Table 3). Even after stratification for ER status, chemotherapy regimen, and type of mutation, no significant association was found between PIK3CA mutation status and response to therapy (Tables 4, 5, 6).

## Discussion

Several lines of *in vitro *evidence suggest that activation status of the PI3K/Akt signaling cascade might alter the chemosensitivity of tumors. For example, in ovarian cancer, overexpression of constitutively active Akt in ovarian cancer cell lines rendered them more resistant to paclitaxel than cancer cells with a low level of Akt expression [26]. In breast cancer cells, transfection of HER2 into MCF7 cells caused PI3K-dependent activation of Akt, resulting in increased resistance to several chemotherapy drugs, including paclitaxel, doxorubicin, 5-fluorouracil, etoposide, and camptothecin. Selective inhibition of PI3K or Akt activity through transfection with dominant-negative expression vectors increased the sensitivity to chemotherapy agents [16]. Activated Ras can also promote cell proliferation and inhibit apoptosis through activation of the PI3K/Akt pathway. When PI3K or MEK was selectively inhibited in Ras-activated MCF7 breast cancer cells, these cells became increasingly sensitive to paclitaxel, doxorubicin, and 5-fluorouracil [15].

Based on these results, we hypothesized that PIK3CA activating mutations may be associated with lesser chemotherapy sensitivity and more residual cancer after preoperative chemotherapy. We examined PIK3CA mutation status in 140 patients with stage II–III breast cancer and correlated the results with clinical and pathological variables, including response to preoperative chemotherapy. The amount of viable invasive cancer after preoperative chemotherapy is a direct measure of chemotherapy sensitivity and is an established surrogate marker of long-term survival [27]. In particular, individuals with pathological complete (pCR) or near complete (RCB-I) response have excellent rates of survival [20].

We did not find any association between PIK3CA status and response to anthracycline-based or anthracycline-containing and paclitaxel-containing chemotherapies. The frequency of PIK3CA mutations was similar in patients with extremely chemotherapy-sensitive tumors indicated by pCR and those with lesser response (RCB-I or RCB-II) or even with extensive residual cancer (RCB-III). ER-positive and ER-negative tumors represent two molecularly different diseases that differ in clinical behavior as well as in chemotherapy sensitivity [28-30]. We previously suggested that different molecular markers may be associated with response to treatment in these two distinct types of breast cancer [31]. For example, high expression of proliferation-related and genomic grade-related genes is associated with chemotherapy sensitivity in both ER-negative and ER-positive tumors. However, expression of genes involved in the E2F3 pathway is associated with increased chemotherapy sensitivity among ER-negative tumors only, whereas a mutant p53 signature and the expression of ER-related genes are associated with lower chemotherapy sensitivity in ER-positive breast tumors [31]. We therefore examined whether the effect of PIK3CA mutation on response to chemotherapy is different among ER-negative and ER-positive tumors. We found no evidence that PIK3CA mutation is predictive of response in either ER-positive or ER-negative tumors.

It was recently reported that PIK3CA mutations in different exons may carry different prognostic values. In one study, exon 9 mutations correlated with unfavorable prognosis (that is, early recurrence and death); in contrast, exon 20 mutations were associated with favorable prognosis [25]. We therefore also examined the association between PIK3CA mutation status and clinical/pathological parameters separately for exon 9 and 20 mutations. We could not detect any difference between response to chemotherapy and PIK3CA mutation type. These observations do not exclude the possibility that assessment of the activity of the PI3K pathway with other more comprehensive protein or mRNA profile-based methods will show predictive value to these or other drugs. PI3K can be activated through many mechanisms other than mutations, and loss of negative feedback loops such as inactivation of PTEN (phosphatase and tensin homolog deleted on chromosome 10) can also activate this complex pathway [32]. Evaluation of other methods to assess PI3K activity to determine its potential predictive value requires further studies.

The sample size of this study is too small to allow for robust analysis of multiple subsets defined by various combinations of ER status, PIK3CA mutation type, and treatment regimen. Stratification for any of these three variables could be done only one at a time. Much larger studies will be needed to address the predictive value of PIK3CA mutations in different molecular subsets of breast cancer in the context of different chemotherapies.

Among the various routine clinical and pathological characteristics that were examined, only nodal status was found to be significantly associated with PIK3CA mutation. Patients with PIK3CA mutations more frequently had node-negative tumors compared with patients with the wild-type gene (52% versus 25%; *P *= 0.012). After adjustment for ER expression, only patients with ER-positive tumors showed this inverse relationship between PIK3CA mutation and nodal status. Furthermore, this correlation was limited to patients harboring exon 9 mutations only. This mutation was significantly more frequent among patients with node-negative disease (66.7% versus 24.8%; *P *= 0.023). The median follow-up for these cases is short; therefore, no survival analysis can be performed currently to examine the prognostic value of PIK3CA mutation in these data.

## Conclusion

In this study, we did not find any evidence that PIK3CA mutations are associated with chemotherapy sensitivity in human breast cancer treated with anthracycline or anthracycline and paclitaxel preoperative chemotherapies. This lack of association between pathological response and mutation status held true for both ER-positive and ER-negative tumors.

## Abbreviations

ER = estrogen receptor; FAC = 5-fluoruracil, doxorubicin, and cyclophosphamide; FEC = 5-fluoruracil, epirubicin, and cyclophosphamide; FISH = fluorescence *in situ *hybridization; HER2 = human epidermal growth receptor 2; IHC = immunohistochemistry; MDACC = M. D. Anderson Cancer Center; mTOR = mammalian target of rapamycin; pCR = pathological complete response; PCR = polymerase chain reaction; PI3K = phosphoinositol 3-kinase; PIK3CA = phosphatidylinositol 3-kinase, catalytic, alpha polypeptide; PR = progesterone receptor; RCB = residual cancer burden; RD = residual disease; TFAC = paclitaxel followed by 5-fluoruracil, doxorubicin, and cyclophosphamide; TFEC = paclitaxel followed by 5-fluoruracil, epirubicin, and cyclophosphamide.

## Competing interests

The authors declare that they have no competing interests.

## Authors' contributions

CL was responsible for data analysis and drafting the manuscript. LC was responsible for data collection and experimental realization and participated in drafting the manuscript. AT was responsible for collection and processing of specimens. KY was responsible for statistical evaluation of the experimental data. HLG, LJBF, VV, and EAS were responsible for specimen and data collection. REH was responsible for collection of clinical data. WFS was responsible for the study design, specimen and data collection, and data evaluation. GNH was responsible for data analysis and critically revised the manuscript. AB was responsible for the study design, data collection, and experimental realization and participated in drafting the manuscript. LP was responsible for the study design, data collection, and drafting/finalizing the manuscript. All authors read and approved the final manuscript
